# *Breaks* no Tempo em Comportamento Sedentário e Marcadores Cardiometabólicos em Adolescentes

**DOI:** 10.36660/abc.20200047

**Published:** 2021-08-09

**Authors:** Natália Maria Mesquita de Lima Quirino, Alcides Prazeres, Arthur Oliveira Barbosa, Gerfeson Mendonça, José Cazuza de Farias

**Affiliations:** 1 Faculdades Nova Esperança João PessoaPB Brasil Faculdades Nova Esperança (FACENE), João Pessoa, PB – Brasil; 2 Grupo de Estudos e Pesquisas em Epidemiologia da Atividade Física João PessoaPB Brasil Grupo de Estudos e Pesquisas em Epidemiologia da Atividade Física (GEPEAF), João Pessoa, PB – Brasil; 3 Educação Física João PessoaPB Brasil Programa Associado de Pós-Graduação em Educação Física (UPE/UFPB), João Pessoa, PB – Brasil; 4 Centro Universitário Cesmac MaceióAL Brasil Centro Universitário Cesmac, Maceió, AL - Brasil; 5 Universidade Federal da Paraíba João PessoaPB Brasil Universidade Federal da Paraíba (UFPB), João Pessoa, PB - Brasil

**Keywords:** Adolescente, Sedentarismo, Adiposidade, Marcadores Cardiometabólicos, Pressão Arterial, Colesterol, Glicose, Triglicérides, Comportamento Sedentário

## Abstract

**Fundamento:**

Interrupções no tempo despendido em comportamento sedentário (*breaks*) têm sido associadas a melhores níveis de indicadores cardiometabólicos na população adulta. No entanto, em adolescentes, os achados sobre essa associação ainda são conflitantes.

**Objetivos:**

Analisar a associação do número de *breaks* por dia em comportamento sedentário com marcadores cardiometabólicos e avaliar se ela é moderada pelo estado nutricional e o tempo excessivo em comportamento sedentário em adolescentes.

**Métodos:**

Estudo transversal com 537 adolescentes (52,3% do sexo feminino), de 10 a 14 anos de idade, de escolas públicas de João Pessoa (PB). O número diário de *breaks* em comportamento sedentário (>100 *counts*/minutos) foi mensurado por meio de acelerômetros (*Actigraph* GT3X+). Os marcadores cardiometabólicos analisados foram: pressão arterial sistólica e diastólica (mmHg), glicose de jejum, colesterol total, triglicerídeos, HDL-c, LDL-c (todos em mg/dL) e índice de massa corporal (IMC) (kg/m^2^). Utilizou-se a regressão linear para analisar a associação do número de *breaks* com marcadores cardiometabólicos e avaliar se ela é moderada pelo estado nutricional e o tempo excessivo em comportamento sedentário. O nível de significância de p<0,05 foi adotado para todas as análises.

**Resultados:**

O número de *breaks* por dia se associou negativamente ao IMC (ß = −0,069; IC95%: −0,102; −0,035), mas não aos demais marcadores cardiometabólicos, e essa associação não foi moderada pelo estado nutricional dos adolescentes (p=0,221) e nem pelo tempo excessivo em comportamento sedentário (p=0,176).

**Conclusão:**

A inclusão de *breaks* no tempo em comportamento sedentário parece contribuir para valores mais baixos do IMC em adolescentes.

## Introdução

Tem sido postulado que o tempo despendido pelos adolescentes em comportamento sedentário – atividades realizadas na posição sentada, reclinada ou deitada, com gasto energético <1,5 METs^1^ – pode representar um fator de risco para alterações desfavoráveis em marcadores cardiometabólicos^2^^,^^3^ e a qualidade de vida relacionada à saúde.^4^ Em função disso, o número de estudos sobre comportamento sedentário e marcadores cardiometabólicos em adolescentes vem crescendo desde a última década.^5^^,^^6^

Os efeitos do comportamento sedentário sobre os marcadores cardiometabólicos podem estar relacionados à diminuição da atividade da enzima lipoproteína lipase (LPL), causada pela hipotensão muscular, decorrente da permanência prolongada na posição sentada ou reclinada.^7^ A menor ação da LPL prejudica a captação de triglicerídeos, glicose, insulina e a síntese de lipoproteína de alta densidade (HDL-C).^8^^,^^9^ Além disso, o tempo despendido nesses comportamentos está associado à redução na prática de atividade física, sobretudo de intensidade leve,^10^ diminuição do gasto energético total diário,^11^ aumento dos indicadores de gordura corporal^2^ e consumo de alimentos ultraprocessados.^2^^,^^12^^,^^13^

Estima-se que adolescentes permanecem cerca de 10 horas por dia em comportamento sedentário.^14^^,^^15^ e 30,2% destes passam mais de 8 horas por dia nesse comportamento.^16^ Nesse sentido, a inclusão de interrupções no tempo despendido por dia nesses comportamentos, denominadas *breaks*, vem sendo considerada como uma das formas de minimizar os efeitos deletérios decorrentes da exposição excessiva e ininterrupta aos comportamentos sedentários.^17^

A incorporação de *breaks* no tempo sedentário reduz a hipotensão muscular,^18^ elevando a atividade da LPL.^19^ Os *breaks* também promovem uma elevação no gasto energético total diário devido ao aumento no tempo de prática de atividade física, sobretudo de intensidade leve,^20^ que pode contribuir para um menor acúmulo de gordura corporal^21^ e melhora nas concentrações de lipoproteínas.^22^

Em adultos, o número de *breaks* por dia tem sido relacionado com redução na glicemia pós-prandial,^21^ perfil lipídico,^23^ índice de massa corporal (IMC)^24^ e controle da adiposidade.^21^ Em adolescentes, a quantidade de estudos sobre *breaks* e marcadores cardiometabólicos ainda é relativamente baixa e com resultados divergentes.^2^^,^^5^^–^^7^^,^^15^^,^^25^^–^^28^ Os estudos que identificaram associações significativas entre *breaks* e marcadores cardiometabólicos nessa população não ajustaram as análises pelo tempo de sono e consumo alimentar;^15^^,^^26^^,^^28^ foram realizados com adolescentes com excesso de peso^27^ ou histórico familiar de obesidade^26^ e não avaliaram se essa associação era moderada pelo estado nutricional^28^ e/ou tempo excessivo em comportamento sedentário.^15^^,^^26^^,^^28^

Outra lacuna de conhecimento é se a associação entre o número de *breaks* e marcadores cardiometabólicos é moderada pelo estado nutricional e/ou tempo sedentário, tendo em vista que o excesso de peso corporal^29^^,^^30^ e o tempo excessivo de comportamento sedentário^2^^,^^6^^,^^7^ estão associados a alterações nos marcadores cardiometabólicos. Dessa forma, a associação entre a realização de *breaks* no tempo em comportamento sedentário e marcadores cardiometabólicos pode ter diferenças (significância e/ou magnitude) conforme o estado nutricional e/ou do tempo despendido em comportamento sedentário. Sendo assim, este estudo analisou a associação do número de *breaks* por dia em comportamentos sedentários com marcadores cardiometabólicos, e se era moderada pelo estado nutricional e tempo excessivo em comportamento sedentário em adolescentes.

## Métodos

Estudo transversal que analisou dados referentes ao primeiro ano (2014) do Estudo Longitudinal sobre Comportamento Sedentário, Atividade Física, Hábitos Alimentares e Saúde de Adolescentes (LONCAAFS). A população de referência foi composta por adolescentes de ambos os sexos, de 10 a 14 anos de idade, que estavam matriculados no 6º ano em escolas da rede pública de ensino da cidade de João Pessoa, Paraíba, Nordeste, Brasil. O Estudo LONCAAFS foi aprovado pelo Comitê de Ética em Pesquisa com Seres Humanos do Centro de Ciências da Saúde da Universidade Federal da Paraíba (Protocolo 240/13).

**Figura 1:**
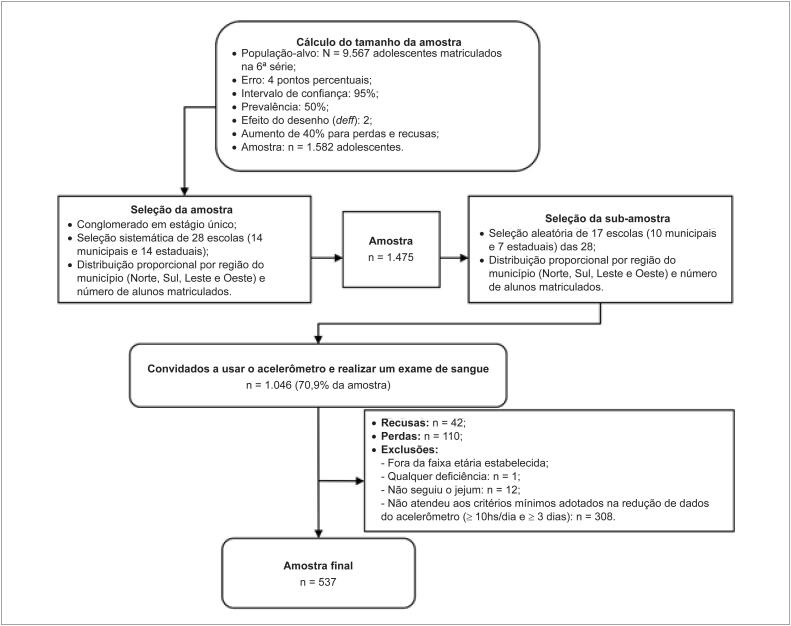
Fluxograma do processo de amostragem do estudo

No presente estudo, foram analisados dados de uma subamostra de adolescentes do Estudo LONCAAFS que utilizou acelerômetros e realizou exame de sangue. Essa escolha foi devido ao número de acelerômetros disponíveis (n = 64), tempo disponível para a coleta de dados e limitação de recursos financeiros. A distribuição da amostra e subamostra levou em consideração a localização da escola na região geográfica no município e o número de alunos matriculados, sendo similar à observada na população de referência. Informações sobre o processo de amostragem deste estudo estão apresentadas em detalhes na Figura 1.

A coleta de dados foi realizada no período de fevereiro a junho e de agosto a dezembro de 2014, por equipe treinada, que utilizou um protocolo uniforme de coleta. Foi utilizado um questionário para avaliar variáveis sociodemográficas, horas de sono e consumo alimentar, aplicado em forma de entrevista “face a face”. Para coletar os dados sociodemográficos, foram considerados sexo (masculino e feminino), idade em anos completos, cor da pele (parda/morena; preta; branca; amarela; indígena, reagrupadas em “branco” e “não branco”), classe econômica (critérios da Associação Brasileira das Empresas de Pesquisa [ABEP], que agrupa as famílias em A1, A2, B1, B2, C1, C2, D e E, posteriormente reagrupada em classe A/B [classe mais alta] e classe C/D/E [classe mais baixa])^31^ e escolaridade da mãe (fundamental incompleto, fundamental completo e médio completo ou mais).

As horas de sono foram mensuradas pela seguinte questão: “nos dias de semana e de fim de semana, a que horas você vai dormir e a que horas você acorda?”. Para determinar o tempo de sono diário, utilizou-se a média ponderada (horas/dia), conforme segue: somatório do valor da diferença entre o horário de dormir e de acordar, multiplicado por cinco para os dias de semana, e por dois para os dias de final de semana, dividido por sete. Essa questão apresentou elevado nível de reprodutibilidade (coeficiente de correlação intraclasse [CCI] = 0,91; IC95%: 0,88 – 0,93).

O consumo de alimentos foi mensurado por meio da aplicação de um recordatório alimentar de 24 horas.^32^ Os adolescentes informaram os alimentos e bebidas consumidos no dia anterior à entrevista, bem como seu meio de preparo, peso e o tamanho das porções. Foi realizada uma replicação em 30% da amostra para aumentar precisão das estimativas de ingestão dietética.^33^ Os dados foram tabulados no *software* “Virtual Nutri”, e o valor calórico total da dieta foi analisado pela equação da *Foodand Nutrition Board of Washinghton.*^34^ Neste estudo, foram utilizados os valores de consumo de lipídios (gramas), total de gorduras saturadas (g), colesterol (mg), sódio (mg) e fibras (g).

A medida de massa corporal foi aferida em balança digital, com precisão de 100 gramas, da marca Techline®, e a estatura realizada em estadiômetro portátil da marca Sanny®. As medidas foram tomadas em triplicata pelo mesmo avaliador e, para fins de resultado final, adotou-se o valor médio das três medidas. O estado nutricional foi determinado por meio do IMC (massa corporal [kg] / estatura [m]^2^) e foi classificado de acordo com os critérios da *World Health Organization* (WHO) em baixo peso, peso normal, sobrepeso e obesidade.^35^

As amostras de sangue foram coletadas por técnicas em enfermagem, no período da manhã, e todos os adolescentes seguiram um jejum de, pelo menos, 12 horas. As concentrações de glicose (mg/dL), triglicerídeos (mg/dL), colesterol total (mg/dL) e lipoproteína de alta densidade (HDL-c; mg/dL) foram determinadas a partir do analisador bioquímico automático Labmax 240 *premium*, do fabricante Labtest, e determinadas pelo método de turbidimetria. A lipoproteína de baixa densidade (LDL-c) foi estimada pela equação de Friedewald, Levy e Fredrickson.^36^

A pressão arterial foi mensurada por meio do monitor automático da marca Omron HEM® – 7200, em uma única visita, no braço direito, com os adolescentes na posição sentada e após 5 minutos de repouso. Esse instrumento apresentou satisfatórios níveis de validade em amostra de adolescentes com faixa etária similar à do presente estudo.^37^ Foram realizadas três medidas (pressão sistólica – CCI = 0,90; IC95%: 0,89 – 0,91 e diastólica – CCI = 0,80; IC95%: 0,78 – 0,82), com intervalo de um minuto entre elas e utilizado o valor médio como resultado final.

O tempo em comportamento sedentário e atividade física moderada a vigorosa e o número de *breaks* foram mensurados por acelerômetros GT3X+ da ActiGraph®. Os adolescentes foram orientados a utilizá-lo durante 7 dias consecutivos, fixado à cintura por um cinto elástico, no lado direito, retirando-o apenas para dormir, tomar banho, realizar atividades de lutas com quedas e aquáticas. A redução dos dados foi realizada no programa *ActiLife* 6.12 e foram adotados os seguintes critérios:^38^ *epoch* de 15 segundos (reintegrados a 60 segundos); tempo de não uso >60 minutos consecutivos de *counts* iguais a zero; uso por, no mínimo, 10 horas por dia, durante 3 dias ou mais dias, sendo pelo menos um de fim de semana.

O tempo em comportamento sedentário e de atividade física de intensidade moderada a vigorosa foi determinado com base nos limiares de Evenson et al.:^38^ ≤100 e >2.295 *counts*/minuto, respectivamente. Um *break* foi operacionalmente definido como o número de ocasiões em que o acelerômetro registrou 100 *counts* ou mais, por pelo menos um minuto.^39^

O número médio de *breaks* por dia foi determinado como segue: número médio de *breaks* em dias de semana (segunda a sexta-feira), multiplicado por cinco, e em dias de final de semana (sábado e domingo), multiplicado por dois, dividindo-se o somatório desses valores por sete. Esse mesmo procedimento foi aplicado para estimar a média ponderada do tempo de atividade física moderada a vigorosa e comportamento sedentário.

A exposição simultânea ao comportamento sedentário e ao número diário de *breaks* foi operacionalizada da seguinte maneira: a) tempo em comportamento sedentário categorizado em <8 horas/dia e ≥8 horas/dia (tempo excessivo em comportamento sedentário) – este ponto de corte foi adotado por ter sido associado a piores indicadores de saúde cardiometabólica em adultos^40^ e não existir um ponto de corte bem estabelecido para adolescentes; b) número de *breaks* por dia em <100 *breaks*/dia e ≥100 *breaks*/dia. Tal classificação foi estabelecida de acordo com curvas ROC [*Receiver Operating Characteristic Curve*], tendo em vista que não existe um ponto de corte definido para o número de *breaks* que demonstre maior risco ou proteção à saúde cardiometabólica, e pelo fato de que a quantidade de 100 *breaks* por dia apresentou valores mais equilibrados de sensibilidade e especificidade. Com base nisso, foram formados quatro grupos de adolescentes: 1) ≥8 horas de comportamento sedentário e <100 *breaks*/dia; 2) ≥8 horas de comportamento sedentário e ≥100 *breaks*/dia; 3) <8 horas de comportamento sedentário e <100 *breaks*/dia; 4) <8 horas de comportamento sedentário e ≥100 *breaks*/dia.

Foram considerados como perda amostral os adolescentes que não retornaram o termo de consentimento livre e esclarecido ou que se ausentaram da escola em pelo menos três visitas para a coleta dos dados. Os critérios de exclusão adotados foram: adolescentes que estavam fora da faixa etária de interesse do estudo (abaixo de 10 e acima de 14 anos); tinham alguma deficiência que os impedissem ou limitassem de praticar atividade física e/ou de responder ao questionário; não atenderam aos critérios adotados na redução de dados do acelerômetro; e relataram não ter seguido o jejum de pelo menos 12 horas.

### Análise estatística

Para descrever as variáveis contínuas, foi utilizada a média e o desvio padrão naquelas com distribuição normal, a mediana e o intervalo interquartil para as que não apresentaram distribuição normal; e distribuição de frequência absoluta (n) e relativa (%) para as categóricas. Foi utilizado o teste de Kolmogorov-Smirnov para verificar se os dados tinham aderência à distribuição normal. Para as variáveis categóricas, foi utilizado o teste do Qui-quadrado e, nas contínuas, os testes T de Student para amostras independentes (variáveis com distribuição normal) e U de Man-Whitney (variáveis sem distribuição normal) para comparar as variáveis entre os adolescentes incluídos e os excluídos das análises.

A regressão linear bruta e ajustada foi utilizada para analisar as associações entre o número de *breaks* por dia em comportamentos sedentários e marcadores cardiometabólicos e se elas eram moderadas pelo estado nutricional e o tempo excessivo em comportamento sedentário. Foram criados modelos de análise para cada variável dependente: glicose (mg/dL); colesterol total (mg/dL); triglicerídeos (mg/dL); HDL-c (mg/dL), LDL-c (mg/dL), pressão arterial sistólica (mmHg), diastólica (mmHg) e IMC (kg/m^2^). Em todos os modelos de análise, a variável independente foi o número médio de *breaks* diário em comportamento sedentário.

As covariáveis analisadas foram: sexo (masculino = 0 e feminino = 1); idade (em anos); classe econômica (A/B = 0 e C/D/E = 1); cor da pele (branco = 0 e não branco = 1); escolaridade da mãe (fundamental incompleto = 0, fundamental completo = 1 e médio completo ou mais = 2); horas de sono (horas/dia); consumo de lipídios (g), total de gorduras saturadas (g), colesterol (mg), sódio (mg) e fibras (g); tempo de uso do acelerômetro (minutos/dia) e de atividade física de intensidade moderada-vigorosa (minutos/dia), de comportamento sedentário (minutos/dia) e IMC, exceto quando esta variável foi tratada como um marcador cardiometabólico no modelo foi tratado como variável dependente.

O método de seleção para entrada das variáveis no modelo ajustado foi o *Foward,* permanecendo aquelas que contribuíram para a redução nos valores dos resíduos, aumentaram o valor de R^2^ ajustado do modelo e modificaram em pelo menos 10% os valores dos coeficientes beta da regressão da variável número de *breaks* por dia. A qualidade de ajuste dos modelos foi avaliada a partir dos valores do fator de inflação de variância (VIF) (valores <5 indicaram ausência de multicolinearidade), distribuição dos resíduos em forma gráfica e homogeneidade das variâncias dos mesmos (Teste de Cook-Weisberg, valores de p >0,05 indicaram presença de homocedasticidade).

Para testar a possível moderação do IMC e do comportamento sedentário na associação entre número de *breaks* por dia e os marcadores cardiometabólicos, foram criados os seguintes termos de interação: a) número de *breaks*/dia*comportamento sedentário (<8 horas e ≥8 horas); b) número de *breaks*/dia*IMC (“sem excesso de peso” e “com excesso de peso corporal”). Esses termos foram incluídos nos modelos ajustados e considerados como interação presente quando o valor de p foi <0,05. Neste caso, os modelos serão tratados separadamente de acordo com a classificação do comportamento sedentário (<8 horas e ≥8 horas) e do IMC (“sem excesso de peso” [baixo peso + peso normal] e “com excesso de peso corporal” [sobrepeso + obesidade]).

Para comparar os valores médios de cada marcador cardiometabólico entre a exposição combinada a comportamento sedentário (<8 horas e ≥8 horas) e número diário de *breaks* (<100 *breaks*/dia e ≥100 *breaks*/dia), foi utilizado o teste de Wald. Nesta análise, foram consideradas as médias de cada marcador cardiometabólico ajustadas pelas mesmas covariáveis dos modelos de regressão. As análises estatísticas foram realizadas no *Stata* 14.0 e o nível de significância adotado foi de p<0,05.

## Resultados

Foram analisados dados de 537 adolescentes de 10 a 14 anos de idade (perdas, recusas e exclusões totalizaram 509 casos [48,6% dos convidados] – Figura 1). O cálculo realizado *a posteriori* indicou que, com um tamanho de efeito (e*ffect size*) igual ou superior a 0,05, alfa (α) de 5% e até 12 preditores no modelo, a amostra do presente estudo teve poder igual a 86%.

Observou-se que não houve diferenças significativas (p≥0,05) para as variáveis sexo, faixa etária, classe econômica, escolaridade da mãe e estado nutricional entre adolescentes que fizeram parte da amostra e da subamostra (dados não apresentados em tabela). Na comparação entre as características dos adolescentes incluídos e excluídos das análises, observou-se maior proporção de adolescentes entre 12 a 14 anos de idade, mães de menor escolaridade, com menores valores de *breaks* por dia, tempo em comportamento sedentário, menor consumo de gordura saturada, maior de lipídios e de sódio nos adolescentes excluídos das análises. Para as demais variáveis, não foram identificadas diferenças significativas (p≥0,05) - Tabela 1.

**Tabela 1:** Comparação das características sociodemográficas, estado nutricional, consumo alimentar, horas de sono, marcadores cardiometabólicos, prática de atividade física, comportamento sedentário e número de *breaks* dos adolescentes incluídos e excluídos da análises (João Pessoa, Paraíba, 2014)

		Incluídos nas análises		Excluídos das análises		p*
Variáveis		(n = 537)		(n = 472)		
		n	%	n	%	
**Sexo**						0,281
Masculino		256	47,7	209	44,3	
Feminino		281	52,3	263	55,7	
**Faixa etária**						<0,001
10-11 anos		344	64,1	230	51,3	
12-14 anos		193	35,9	242	48,7	
**Classe econômica**						0,614
A/B		170	36,3	144	34,7	
C/D/E		298	63,7	271	65,3	
**Cor da pele** §						0,352
Branca		16	20,8	87	18,6	
Não branca		61	79,2	382	81,4	
**Escolaridade da mãe** //						0,010
Fundamental incompleto		148	33,5	166	41,9	
Fundamental completo		130	29,4	119	30,1	
Médio completo ou mais		164	37,1	111	28,0	
**Índice de massa corporal (IMC)**						0,085
Baixo peso		14	2,6	14	3,0	
Peso normal		326	61,4	321	68,6	
Sobrepeso		115	21,7	83	17,7	
Obesidade		76	14,3	50	10,7	
**Exposição a comportamento sedentário**						
<8 horas/dia		343	63,9	192	66,9	0,386
≥8 horas/dia		194	36,1	95	33,1	
	**n**	**Média**	**DP**	**Média**	**DP**	**p** †
**Variáveis comportamentais**						
Horas de sono (horas/dia)§	536	9,7	1,6	9,6	1,6	0,871
Número de *breaks* (número/dia)¶	537	100,3	91,5-108,3	92,0	82,5-104,0	<0,001‡
Atividade física (minutos/dia)¶	537	29,1	17,9-45,1	30,5	16,5- 47,0	0,710‡
Comportamento sedentário (minutos/dia)¶	537	451,0	392,7-513,1	432,8	377,0-500,7	0,022‡
Uso do acelerômetro (minutos/dia)	537	855,3	94,9	816,0	109,7	<0,001
**Consumo alimentar**						
Lipídio (g)	528	71,4	45,4	77,7	51,5	0,044
Gordura saturada (g)¶	528	15,0	8,0-23,0	17,0	10,0-26,0	0,001‡
Sódio (mg)¶	528	2.055,5	1.420,5-2.852,0	2.161,0	1.534,0-3.053,0	0,028‡
Fibras (g)	528	23,1	14,2	24,3	14,4	0,198
Colesterol (mg)	528	176,8	190,4	188,5	240,2	0,397
**Marcadores cardiometabólicos**						
IMC (kg/m^2^)	531	19,5	4,0	19,5	3,6	0,410
PAS (mmHg)	537	105,8	9,5	105,2	8,6	0,321
PAD (mmHg)	537	62,4	7,0	61,9	6,9	0,318
Glicose (mg/dL)	537	91,1	10,2	91,4	23,1	0,819
Colesterol (mg/dL)§	536	159,4	31,7	158,1	32,1	0,580
Triglicerídeo (mg/dL) ¶	534	75,0	56-102	73,0	54-98	0,516‡
HDL (mg/dL)§	536	43,9	9,5	43,4	9,3	0,463
LDL (mg/dL)§	536	98,3	28,2	98,2	28,5	0,945

DP: desvio padrão;

* teste qui-quadrado;

† T de Student para amostras independentes;

‡ teste U de Mann-Whitney;

§ variáveis com menor número de perdas (n = 1);

// variável com maior número de perdas (n = 101);

¶ dados apresentados como mediana e intervalo interquartil.

A maioria dos adolescentes era do sexo feminino, de 10 a 11 anos de idade, com cor da pele não branca, pertencia à classe econômica C/D/E, filhos de mães com pelo menos o ensino fundamental completo e pouco mais de um terço tinha excesso de peso corporal. O tempo de atividade física, comportamento sedentário e número de *breaks* despendidos pelos adolescentes foi de respectivamente, 29,1; 451,0 e 100,3 (Tabela 1).

No modelo bruto, houve associação significativa entre número médio de *breaks* por dia e LDL-c (p = 0,030), pressão arterial sistólica (p = 0,006) e IMC (p <0,001). Na análise ajustada, apenas associação entre o número médio de *breaks* por dia e o IMC (p <0,001) permaneceu estatisticamente significativa. O comportamento sedentário e o IMC não moderaram a associação entre número de *breaks* por dia e marcadores cardiometabólicos (Tabela 2). Os modelos finais alcançaram boa qualidade de ajuste: ausência de multicolinearidade (VIF entre 1,03 e 3,39), presença de homocedasticidade (teste de Cook-Weisberg com valores de p variando de 0,054 a 0,335) e distribuição normal nos resíduos da regressão.

**Tabela 2:** Regressão linear bruta e ajustada para associação entre o número médio de *breaks* por dia e marcadores cardiometabólicos em adolescentes de João Pessoa, Paraíba (2014)

Variáveis		Bruta			Ajustada†			Termo de interação comportamento sedentário‡			Termo de interação IMC§	
	ß	(IC95%)	p	ß	(IC95%)	p	ß	(IC95%)	p	ß	(IC95%)	p
Glicose (mg/dL)	0,011	-0,052; 0,073	0,737	-0,039	-0,138; 0,061	0,446	-0,021	-0,050; 0,009	0,176	-0,003	-0,037; 0,031	0,865
Colesterol (mg/dL)	-0,181	-0,375; 0,013	0,067	-0,042	-0,336; 0,251	0,778	0,037	-0,051; 0,126	0,404	-0,046	-0,146; 0,055	0,371
Triglicerídeos (mg/dL)	-0,001	-0,004; 0,002	0,539	0,000	-0,004; 0,005	0,861	0,001	-0,001; 0,002	0,285	0,001	-0,001; 0,002	0,248
HDL-c (mg/dL)	0,040	-0,018; 0,099	0,174	-0,016	-0,104; 0,073	0,729	-0,013	-0,041; 0,014	0,341	0,005	-0,027; 0,036	0,761
LDL-c (mg/dL)	-0,190	-0,362; -0,018	0,030	0,019	-0,238; 0,277	0,882	0,021	-0,058; 0,101	0,598	-0,057	-0,149; 0,034	0,221
PAS (mmHg)	-0,081	-0,139; -0,023	0,006	-0,011	-0,090; 0,068	0,778	0,017	-0,008; 0,041	0,176	-0,005	-0,033; 0,023	0,710
PAD (mmHg)	-0,029	-0,072; 0,014	0,184	-0,026	-0,088; 0,037	0,421	0,008	-0,011; 0,027	0,387	-0,004	-0,025; 0,018	0,739
IMC (kg/m^2^)	-0,051	-0,075; -0,026	0,000	-0,069	-0,102; -0,035	0,000	-0,004	-0,014; 0,006	0,440	--	--	--

ß: coeficiente beta; IC95%: intervalo de confiança de 95%; HDL-c: lipoproteína de alta densidade; LDL-c: lipoproteína de baixa densidade; PAS: pressão arterial sistólica; PAD: pressão arterial diastólica; IMC: índice de massa corporal;

† modelo ajustado por sexo, idade, cor da pele, classe econômica, escolaridade da mãe, horas de sono (horas/dia), consumo de fibra (g), lipídios (g), gordura saturada (g), colesterol (mg), sódio (mg), tempo de uso de acelerômetro (min/dia), tempo total de atividade física moderada a vigorosa (min/dia), tempo total de comportamento sedentário (min/dia) e IMC (kg/m^2^), exceto quando esta variável foi tratada como um marcador cardiometabólico no modelo for tratado como variável dependente;

‡ modelo ajustado + termo de interação entre comportamento sedentário (<8 horas/dia vs. ≥8 horas/dia) e número de breaks/dia;

§ modelo ajustado + termo de interação entre IMC (kg/m^2^) e número de breaks/dia.

**Figura 2:**
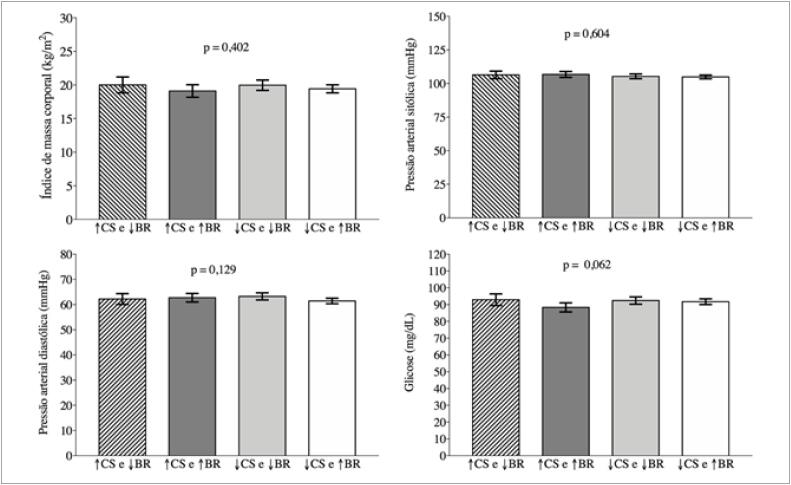
Comparação dos valores médios de IMC, pressão arterial sistólica e diastólica e glicose entre exposição combinada a comportamento sedentário e breaks em adolescentes (João Pessoa, Paraíba, 2014). ↑CS = ≥8 horas/dia; ↓CS = <8 horas/dia; ↑BR = ≥100 breaks/dia; ↓BR = <100 breaks/dia. CS: comportamento sedentário; BR: breaks.

Os resultados do teste de Wald indicaram não existir diferenças significativas nos valores médios dos marcadores cardiometabólicos entre os adolescentes expostos a ≥8 horas de comportamento sedentário e <100 *breaks*/dia, ≥8 horas de comportamento sedentário e ≥100 *breaks*/dia, <8 horas de comportamento sedentário e <100 *breaks*/dia e <8 horas de comportamento sedentário e ≥100 *breaks*/dia (Figuras 2 e 3).

**Figura 3:**
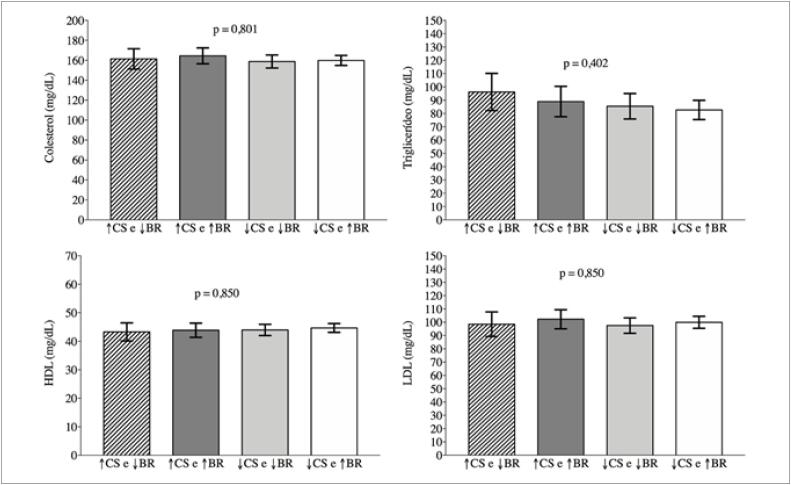
Comparação dos valores médios de colesterol, triglicerídeos, HDL e LDL entre a exposição combinada a comportamento sedentário e breaks em adolescentes (João Pessoa, Paraíba, 2014). ↑CS = ≥8 horas/dia; ↓CS = <8 horas/dia; ↑BR = ≥100 breaks/dia; ↓BR = <100 breaks/dia; CS: comportamento sedentário; BR: breaks.

## Discussão

Os resultados deste estudo indicaram que os adolescentes com maior número de *breaks* por dia no tempo em comportamento sedentário apresentaram valores mais baixos de IMC. Contudo, as associações com os demais marcadores cardiometabólicos não foram significativas e nem moderadas pelo estado nutricional e tempo excessivo em comportamento sedentário.

Estudos com adultos demonstraram que maior ocorrência de *breaks* estava associada à redução dos efeitos deletérios à saúde cardiometabólica decorrentes do tempo despendido em comportamento sedentário.^41^ No entanto, em adolescentes, essa relação vem sendo observada apenas com indicadores de gordura corporal.^2^^,^^6^ A ausência de associação dos *breaks* com os marcadores cardiometabólicos pode estar relacionada ao fato de os adolescentes acumularem a maior parte do seu tempo diário em comportamento sedentário em blocos de até 5 minutos.^1^^,^^14^^,^^16^ A exposição a blocos de tempo curtos em comportamento sedentário pode minimizar a redução da atividade da enzima LPL (lipoproteína lipase) e contribuir para o aumento do gasto energético. Esses dois fatores estão relacionados com diminuição nos níveis sanguíneos de glicose e triglicerídeos e aumento de HDL-c.^42^

O tempo excessivo de comportamento sedentário não moderou a associação entre o número de *breaks* e marcadores cardiometabólicos. Uma análise suplementar demonstrou que mais de 80% do tempo sedentário dos adolescentes do presente estudo foi acumulado em intervalos de tempo inferiores a 10 minutos, mesmo nos que tinham tempo excessivo em comportamento sedentário (dados não apresentados em tabela). Diante disso, é possível que os benefícios da inclusão de *breaks* sobre os marcadores cardiometabólicos sejam observados nos adolescentes expostos a longos períodos ininterruptos de comportamento sedentário.

Alguns estudos experimentais identificaram que a inclusão de *breaks* de intensidade moderada a vigorosa, com 3 minutos de duração e a cada meia hora, durante 3 horas de exposição a comportamentos sedentários, reduziu os níveis de insulina, peptídio C^27^^,^^43^ e de glicose.^43^ No entanto, esse resultado não foi confirmado por Sanders et al.,^1^ ao analisar os efeitos da inclusão de *breaks* de intensidade leve, com duração de 2 minutos e a cada 20 minutos, durante 8 horas de exposição a comportamentos sedentários. A inconsistência nos resultados desses estudos mostra que mais investigações são necessárias para suportar a hipótese de que os benefícios da inclusão de *breaks* ocorreriam em adolescentes expostos a tempos prolongados e ininterruptos de comportamento sedentário.

Uma possível menor resposta da LPL ao efeito hipotensor do comportamento sedentário e uma maior capacidade dos adolescentes para manter os marcadores cardiometabólicos em valores próximos aos considerados normais (homeostase), comparado aos adultos, são outras fontes de explicação para a ausência de associação entre *breaks* e marcadores cardiometabólicos nesse grupo populacional.

No presente estudo, verificou-se que adolescentes que tinham maior número de *breaks* apresentaram menores valores para o IMC, reforçando achados de outros estudos.^2^^,^^6^ Em termos de relevância clínica, o efeito dos *breaks* sobre o IMC foi de baixa magnitude (para cada *break* realizado, estima-se uma diminuição de 0,069 de kg/m^2^ no IMC – *effect size* = 0,076). Apesar disso, a inclusão de *breaks* pode ser uma prática facilmente implementada no contexto de vida do adolescente, podendo ser uma dentre as várias ações a serem utilizadas em intervenções que visem à redução e/ou ao controle do IMC.

A inclusão de *breaks* no tempo sentado tende a promover aumento no gasto energético devido a maior prática de atividade física. Júdice et al.,^15^ em estudo com adultos, observaram que a inclusão de um *break* resultava em aumento médio de 1,49 kcal/min no gasto energético. Nos adolescentes, os *breaks* podem gerar um gasto energético similar ao de adultos. Desse modo, a realização de 100 *breaks* ao longo do dia equivaleria a uma caminhada de cerca de 30 minutos de intensidade moderada.^44^ Maior tempo em comportamento sedentário está relacionado a um menor número de *breaks* e tempo de atividade física no lazer,^45^ e maior consumo de guloseimas, refrigerantes e alimentos industrializados/ultraprocessados.^46^ Desse modo, adolescentes que realizam mais *breaks* durante o dia teriam maior tempo de atividade física no lazer e menor consumo de alimentos ultraprocessados. Por ser um estudo transversal, não se pode descartar a possibilidade de que adolescentes com maior IMC teriam menor movimentação espontânea ao longo dia, resultando em um menor número de *breaks* no comportamento sedentário.

Os pontos fortes deste estudo incluem: análise de dados em uma amostra representativa da população de escolares do sexto ano da rede pública de ensino de uma cidade do Nordeste do Brasil e poder suficiente para testar as hipóteses propostas; análise de diferentes marcadores cardiometabólicos; consideração, nas análises, de fatores de confusão importantes para a relação entre comportamento sedentário e marcadores cardiometabólicos (prática de atividade física, horas de sono, consumo alimentar).

As principais limitações deste estudo foram: ausência de mensuração da maturação sexual dos adolescentes, por ser uma variável que pode influenciar os marcadores cardiometabólicos^47^^,^^48^ e alguns tipos de comportamento sedentário;^49^ reintegração dos dados do acelerômetro de *epochs* de 15 para 60 segundos, o que pode ter subestimado o tempo em comportamento sedentário^50^ e as magnitudes das associações; utilização do acelerômetro para mensurar o número de *breaks* em comportamento sedentário, uma vez que esse instrumento tem como base a medida da aceleração corporal e não tem capacidade de diferenciar de forma acurada a postura corporal (sentado, reclinado, em pé).^51^

## Conclusão

Adolescentes com mais *breaks* por dia no tempo em comportamento sedentário tinham valores médios mais baixos para o IMC, mas não apresentaram diferenças nos valores dos demais marcadores cardiometabólicos bioquímicos (glicose, triglicerídeos, HDL-c, LDL-c, colesterol total e pressão arterial), independentemente do seu estado nutricional e do tempo excessivo de exposição ao comportamento sedentário.
