# Supplementary material for: Breaks no Tempo em Comportamento Sedentário e Marcadores Cardiometabólicos em Adolescentes
Source: Arq Bras Cardiol. 2021 Aug 9;117(2):352–62. [Article in Portuguese] doi: 10.36660/abc.20200047 (PMC8395781; doi:10.36660/abc.20200047)
Supplement: Translation

# Translation: Breaks in Sedentary Time and Cardiometabolic Markers in Adolescents

**Authors:** Natália Maria Mesquita de Lima Quirino, Alcides Prazeres, Arthur Oliveira Barbosa, Gerfeson Mendonça, José Cazuza de Farias

**Affiliations:** 1 João PessoaPB Brazil Faculdades Nova Esperança (FACENE), João Pessoa, PB – Brazil; 2 João PessoaPB Brazil Grupo de Estudos e Pesquisas em Epidemiologia da Atividade Física (GEPEAF), João Pessoa, PB – Brazil; 3 João PessoaPB Brazil Programa Associado de Pós-Graduação em Educação Física (UPE/UFPB), João Pessoa, PB – Brazil; 4 MaceióAL Brazil Centro Universitário Cesmac, Maceió, AL - Brazil; 5 João PessoaPB Brazil Universidade Federal da Paraíba (UFPB), João Pessoa, PB - Brazil

**Keywords:** Adolescent, Sedentarism, Adiposity, Cardiometabolic Markers, Blood Arterial, Cholesterol, Glucose, Triglycerides, Sedentary Behavior

## Abstract

**Background:**

The interruption of the time spent in sedentary behavior (breaks) has been associated with better levels of cardiometabolic indicators in the adult population, but in adolescents, further investigations are still needed to confirm these findings.

**Objectives:**

To analyze the association of the number of breaks per day in sedentary behaviors with cardiometabolic markers and whether it was moderated by nutritional status and excessive time on sedentary behavior in adolescents.

**Methods:**

This is a cross-sectional study of 537 adolescents (52.3% girls), aged between 10 and 14 years, enrolled in public schools in the city of João Pessoa, Paraíba state, Brazil. The number of daily breaks (>100 counts/minutes) in sedentary time was measured by Actigraph GT3X+ accelerometers. The following cardiometabolic markers were analyzed: systolic and diastolic blood pressure (mmHg), fasting blood glucose levels, total cholesterol, triglycerides, HDL-c, LDL-c (all in mg/dL) and body mass index (BMI) (kg/m^2^). Linear regression was used to analyze the association between the number of breaks and cardiometabolic markers and whether this association was moderated by nutritional status and excessive time in sedentary behavior. The significance level of p<0.05 was adopted for all analyses.

**Results:**

The number of daily breaks was negatively associated with BMI (boys – ß = −0.083; 95%CI: −0.132; −0.034 and girls – ß = −0.115; 95%CI: −0.169; −0.061), but not with the remaining cardiometabolic markers. The number of breaks per day was negatively associated with BMI (ß = −0.069; 95% CI: −0.102; −0.035), but not with the other cardiometabolic markers and this association was not moderated by the adolescents' nutritional status (p=0.221), or by excessive time in sedentary behavior (p=0.176).

**Conclusions:**

Including breaks in sedentary time seems to contribute to lower BMI values in adolescents.

## Introduction

It has been hypothesized that the time spent by adolescents in sedentary behavior - activities performed in a sitting, reclining position or lying down., with energy expenditure <1.5 METs^1^ - may be a risk factor for unfavorable changes in cardiometabolic markers^2^^,^^3^ and health-related quality of life.^4^ As such, the number of studies that have analyzed the relationship between sedentary behavior and cardiometabolic markers has increased in the last decade.^5^^,^^6^

The effects of sedentary behavior on cardiometabolic markers may be related to the decreased activity of the enzyme lipoprotein lipase (LPL), caused by muscle hypotension, resulting from prolonged sitting or reclining.^7^ The lower action of LPL impairs the uptake of triglycerides, glucose, insulin and the synthesis of high density lipoprotein (HDL-C).^8^^,^^9^ In addition, the time spent on these behaviors is associated with a reduction in the practice of physical activities, especially those of light intensity,^10^ decrease in the total daily energy expenditure,^11^ increase in body fat indicators^2^ and the consumption of ultra-processed foods.^2^^,^^12^^,^^13^

It is estimated that adolescents spend around 10 hours a day on sedentary behavior,^14^^,^^15^ with 30.2% spending more than eight hours.^16^ In this sense, the inclusion of interruptions during the time spent per day on these behaviors, called breaks, has been considered as one of the ways to minimize the harmful health effects resulting from excessive and uninterrupted exposure to sedentary behaviors.^17^

The incorporation of breaks in sedentary time reduces muscle hypotension,^18^ increasing LPL activity.^19^ The breaks also promote an increase in total daily energy expenditure due to an increase in the time of physical activities, especially those of light intensity,^20^ which can contribute to less accumulation of body fat^21^ and improvement in lipoprotein concentrations.^22^

In adults, the number of breaks per day has been associated with a reduction in postprandial glycemia,^21^ lipid profile,^23^ and body mass index (BMI),^24^ as well as in adiposity control.^21^ In adolescents, the number of studies on breaks and cardiometabolic markers is still relatively low, with divergent results.^2^^,^^5^–^7^,^15^,^25^–^28^ Studies that identified significant associations between breaks and cardiometabolic markers in this population did not adjust the analyses by sleep duration and food consumption,^15^^,^^26^,^28^ were performed with overweight adolescents^27^ or those with a family history of obesity^26^ and did not assess whether this association was moderated by nutritional status^28^ and/or excessive time in sedentary behavior.^15^^,^^26^^,^^28^

Another knowledge gap is whether the association between the number of breaks and cardiometabolic markers is moderated by nutritional status and/or time in sedentary behavior, considering that overweight^29^^,^^30^ and excessive time in sedentary behavior^2^^,^^6^^,^^7^ are associated with changes in cardiometabolic markers. Thus, the association between taking breaks during time spent in sedentary behavior and cardiometabolic markers may differ (regarding significance and/or magnitude) according to the individual's nutritional status and/or the time spent in sedentary behavior. Thus, this study analyzed the association between the number of breaks per day in sedentary behaviors and cardiometabolic markers and whether it was moderated by nutritional status and excessive time in sedentary behavior in adolescents.

## Methods

This cross-sectional research analyzed data from the first year (2014) of the LONCAAFS study (Longitudinal Study on Sedentary Behavior, Physical Activity, Eating Habits and Adolescent Health). The reference population consisted of adolescents of both genders, aged 10 to 14 years, enrolled in 6^th^ grade at public schools in João Pessoa, Paraíba state, Northeastern Brazil. The LONCAAFS study was approved by the Human Research Ethics Committee of the Health Sciences Center at Universidade Federal da Paraíba (Protocol 240/13).

In this study, we analyzed data from a subsample of adolescents from the LONCAAFS study, which used accelerometers and underwent a blood test. This choice was made due to the number of accelerometers available (n = 64), the time available for data collection (school year) and lack of financial resources. The distribution of the sample and subsample in the geographic region of the municipality and the number of students enrolled were similar to that observed in the reference population. Information on sample selection and calculation is presented in details in Figure 1.

**Figure 1:** Flowchart of the study sampling process

Data were collected between February and June and from August to December 2014, by a trained team. A questionnaire in the form of a face-to-face interview was applied to collect the following sociodemographic data: gender (male and female); age, skin color (brown; black; white; yellow; indigenous, reclassified as white and non-white); socioeconomic class [Brazilian Association of Research Companies (ABEP) criteria, which classifies families into classes A1, A2, B1, B2, C1, C2, D and E, later reclassified as class A/B (higher class) and C/D/E (lower class)]^31^ and mother's level of schooling (incomplete elementary school, complete elementary school, complete high school and higher education).

The hours of sleep were measured by the following question: “on weekdays and on the weekend, what time do you go to sleep and what time do you wake up?”. Daily hours of sleep were determined as follows: the difference between bed and wake times during the week multiplied by five, added to the difference between these times on the weekend, multiplied by two. This result was divided by seven in order to obtain the average weighted number of hours of sleep per day. This question showed a high level of reproducibility (intraclass correlation coefficient – ICC = 0.91; 95% CI: 0.88 – 0.93).

Food intake was based on a 24-hour dietary recall.^32^ The adolescents recorded the food items and beverages they had consumed on the day before the interview, as well as the weight and food preparation methods used. Thirty percent of the sample was replicated to increase the accuracy of the estimated food intake.^33^ The data were tabulated in the Virtual Nutri software and the total calorie value was analyzed using the equation created by the Food and Nutrition Board of Washington.^34^ In this study we used lipid and saturated fat (grams), cholesterol (mg), sodium (mg) and fiber (g) values.

BMI was measured with a digital balance, accurate to 100 grams and height was measured with a portable stadiometer. The measures were taken in triplicate by the same rater and the average value was used. Nutritional status was determined by the BMI (BMI = weight [kg] /height [m]^2^) and classified according to the criteria of the World Health Organization (WHO).^35^

The blood samples were collected in the morning by nursing technicians and all the adolescents fasted for at least 12 hours before the collection. Levels of glucose (mg/dL), triglycerides (mg/dL), total cholesterol (mg/dL) and high-density lipoprotein – HDL-c (mg/dL) were determined using a Labmax 240 premium automatic biochemical analyzer (Labtest) and the turbidimetry method. Low-density lipoprotein (LDL-c) was estimated by the Friedewald, Levy and Fredrickson equation.^36^

Blood pressure was measured in the right arm using an Omron HEM – 7200 automatic monitor, at a single visit, with adolescents in the sitting position, after a five-minute rest. This instrument showed satisfactory levels of validity in a sample of adolescents with an age range similar to the present study.^37^ Three measurements were obtained (systolic– intraclass correlation coefficient – ICC = 0.90; 95%CI: 0.89 – 0.91 and diastolic pressure – ICC = 0.80; 95%CI: 0.78 – 0.82), with a one-minute interval between them and the average value was used as the final result.

Time spent on sedentary behavior and moderate to vigorous physical activities and the number of breaks were measured by Actigraph GT3X+ accelerometers. The adolescents were instructed to use the accelerometer for seven consecutive days, attached to the right side of their waist by an elastic belt, removing it only when sleeping, bathing, and engaging in aquatic activities or martial arts involving falls. The accelerometer data were reduced using the ActiLife 6.12 program, adopting the following criteria:^38^ A 15-second epoch (reintegrated to 60 seconds); nonuse time > 60 consecutive minutes of counts equal to zero; used for at least 10 hours a day for three or more days, including at least one weekend.

Sedentary behavior and moderate to vigorous physical activity duration were determined based on the thresholds of Evenson et al.:^38^ ≤ 100 and > 2,295 counts/minute, respectively. A break was operationally defined as the number of times in which the accelerometer recorded 100 counts or more for at least one minute.^39^

The number of daily breaks was determined as follows: average number of daily breaks during the week (Monday to Friday), multiplied by five, and on weekends (Saturday and Sunday), multiplied by two, dividing the sum of these values by seven. This procedure was applied to estimate the weighted mean of time spent in moderate to vigorous physical activity and sedentary behavior.

The simultaneous exposure to sedentary behavior and the daily number of breaks was operationalized as follows: a) time in sedentary behavior categorized as <8 hours/day and ≥8 hours/day (excessive time in sedentary behavior) - this cutoff point was adopted because it was associated with worse cardiometabolic health indicators in adults^40^ and there is no well-established cutoff point for adolescents; b) number of breaks per day as <100 breaks/day and ≥100 breaks/day. This classification was established according to ROC [Receiver Operating Characteristic] curves, considering that there is no defined cutoff point for the number of breaks that demonstrate greater risk or protection regarding cardiometabolic health and the fact that the amount of 100 daily breaks showed more balanced values of sensitivity and specificity. Based on this, four groups of adolescents were created: 1) ≥ 8 hours of sedentary behavior and <100 breaks/day; 2) ≥ 8 hours of sedentary behavior and ≥ 100 breaks/day; 3) <8 hours of sedentary behavior and <100 breaks/day and; 4) <8 hours of sedentary behavior and ≥ 100 breaks/day.

Adolescents who did not provide written informed consent or were absent from school on at least three data collection visits were considered sample losses. The exclusion criteria comprised adolescents outside the established age range (younger than 10 and older than 14 years), any impairment that hindered or limited physical activity and/or prevented them from completing the questionnaire; individuals who did not meet the minimum criteria adopted for accelerometer data reduction and those who did not fast for at least 12 hours.

### Data analysis

To describe the quantitative variables, mean and standard deviation were used for variables with a normal distribution, and median and interquartile range for those that did not have a normal distribution, and absolute (n) and relative (%) frequencies for qualitative ones. The Kolmogorov-Smirnov test was used to verify whether the data showed a normal distribution. The chi-square test was used for the qualitative variables, and for the quantitative ones, Student's *t* test for independent samples (variables with normal distribution) and the Mann-Whitney U test (variables with non-normal distribution) were used to compare the variables between the included adolescents and those excluded from the analysis.

Simple and multiple linear regression was used to analyze the associations between the number of daily breaks in sedentary behavior and cardiometabolic markers and whether they were moderated by the nutritional status and excessive time in sedentary behavior. The analysis models were created for each dependent variable: levels of glucose [mg/dL]; total cholesterol [mg/dL]; triglycerides [mg/dL]; HDL-c [mg/dL], LDL-c [mg/dL]; systolic [mmHg] and diastolic [mmHg] blood pressure and BMI (kg/m^2^).

The covariables analyzed were: gender (male = 0 and female = 1); age (in years); socioeconomic class (A / B = 0 and C / D / E = 1); skin color (white = 0 and not-white = 1); mother's level of schooling (incomplete elementary school = 0, complete elementary school = 1 and complete high school or higher = 2); hours of sleep (hours / day); consumption of lipids (g), total saturated fats (g), cholesterol (mg), sodium (mg) and fibers (g); time using the accelerometer (minutes/day) and physical activity of moderate-vigorous intensity (minutes/day) and sedentary behavior (minutes / day) and BMI, except when this variable was treated as a cardiometabolic marker in the model.

The selection method for entering the variables in the adjusted model was the Forward method, and variables that contributed to the reduction in the residual values, increased the adjusted R^2^ value of the model, modified the values of the beta coefficients of the regression of the model by at least 10% of the variable number of breaks per day remained in the model. The fit quality of the models was assessed based on the values of the variance inflation factor. When assessing the fit quality of the models, the values of the variance inflation factor - VIF - were considered (values <5 indicated absence of multicollinearity), with residuals in graph form and homogeneity of variances (Cook-Weisberg test, p≥0.05 indicates the presence of homoscedasticity).

To test the possible moderation of BMI and sedentary behavior in the association between number of breaks per day and cardiometabolic markers, the following interaction terms were created: a) number of breaks/day*sedentary behavior (<8 hours and ≥8 hours); b) number of breaks/day*BMI (without overweight and with overweight). These terms were included in the adjusted models and considered as a present interaction when the p value was <0.05. In this case, the models will be treated separately according to the classification of sedentary behavior (<8 hours and ≥8 hours) and BMI (without overweight and with overweight).

The Wald test was used to compare the mean values of each cardiometabolic marker between combined exposure to sedentary behavior (<8 hours and ≥8 hours) and daily number of breaks (<100 breaks / day and ≥100 breaks / day). In this analysis, the means of each cardiometabolic marker adjusted by the same covariables of the regression models were considered. Stata 14.0 software was used and the significance level was set at p<0.05.

## Results

The data of 537 adolescents, aged 10 to 14 years were analyzed (losses, refusals and exclusions totaled 509 cases, 48.6% of those invited to participate) – Figure 1. The *a posteriori* calculation indicated that with an effect size equal to or greater than 0.05; alpha (α) of 5%; and up to 12 predictors in the model, the sample of the present study had a power equal to 86%.

There was no significant difference (p≥0.05) for the variables gender, age group, socioeconomic class, mother's level of schooling and nutritional status between the sample and subsample of adolescents (data not shown in table). When comparing the characteristics of the adolescents included and excluded from the analyses, there was a higher proportion of adolescents between 12 and 14 years of age, mothers with a lower level of education, with lower values of breaks per day, time in sedentary behavior, less consumption of saturated fat, higher consumption of lipids and sodium in adolescents who were excluded from the analyses. No significant differences were identified for the other variables (p≥0.05) - Table 1.

**Table 1:** Comparison of the descriptions of sociodemographic characteristics, nutritional status, food consumption, cardiometabolic markers, physical activity, sedentary behavior and number of breaks in the adolescents included and excluded from the analysis, João Pessoa, Paraíba, 2014

		Included in the analyses		Excluded from the analyses		p*
Variables		(n = 537)		(n = 472)		
		n	%	n	%	
**Gender**						0.281
Male		256	47.7	209	44.3	
Female		281	52.3	263	55.7	
**Age**						<0.001
10-11 (years)		344	64.1	230	51.3	
12-14 (years)		193	35.9	242	48.7	
**Socioeconomic class**						0.614
A/B		170	36.3	144	34.7	
C/D/E		298	63.7	271	65.3	
**Skin color** §						0.352
White		16	20.8	87	18.6	
Non-white		61	79.2	382	81.4	
**Mother's level of schooling** //						0.010
Incomplete elementary school		148	33.5	166	41.9	
Elementary school		130	29.4	119	30.1	
Complete high school and higher education		164	37.1	111	28.0	
**Body mass index (BMI)**						0.085
Underweight		14	2.6	14	3.0	
Normal weight		326	61.4	321	68.6	
Overweight		115	21.7	83	17.7	
Obesity		76	14.3	50	10.7	
Exposure to sedentary behavior						
<8 hours/day		343	63.9	192	66.9	0.386
≥8 hours/day		194	36.1	95	33.1	
	**n**	**Mean**	**SD**	**Mean**	**SD**	**p** †
**Behavior variables**						
Sleep hours (hours/day)§	536	9.7	1.6	9.6	1.6	0.871
Numbers of breaks (number/day)¶	537	100.3	91.5-108.3	92.0	82.5-104.0	<0.001‡
Physical activity (minutes/day)¶	537	29.1	17.9-45.1	30.5	16.5- 47.0	0.710‡
Sedentary behavior (minutes/day)¶	537	451.0	392.7-513.1	432.8	377.0-500.7	0.022‡
Accelerometer usage (minutes/day)	537	855.3	94.9	816.0	109.7	<0.001
**Food intake**						
Lipid (g)	528	71.4	45.4	77.7	51.5	0.044
Total saturated fat (g)¶	528	15.0	8.0-23.0	17.0	10.0-26.0	0.001‡
Sodium (mg)¶	528	2.055.5	1.420.5-2.852.0	2.161.0	1.534.0-3.053.0	0.028‡
Fibers (g)	528	23.1	14.2	24.3	14.4	0.198
Cholesterol (mg)	528	176.8	190.4	188.5	240.2	0.397
**Cardiometabolic markers**						
BMI (kg/m^2^)	531	19.5	4.0	19.5	3.6	0.410
SBP (mmHg)	537	105.8	9.5	105.2	8.6	0.321
DBP (mmHg)	537	62.4	7.0	61.9	6.9	0.318
Glucose (mg/dL)	537	91.1	10.2	91.4	23.1	0.819
Cholesterol (mg/dL)§	536	159.4	31.7	158.1	32.1	0.580
Triglycerides (mg/dL) ¶	534	75.0	56-102	73.0	54-98	0.516‡
HDL (mg/dL)§	536	43.9	9.5	43.4	9.3	0.463
LDL (mg/dL)§	536	98.3	28.2	98.2	28.5	0.945

SD: standard deviation;

* chi-square test;

† Student's T for independent variables;

‡ Mann-Whitney U test;

§ Variables with fewer losses (n = 1);

// Variable with more losses (n = 101);

¶ Data presented as median and interquartile range. BMI: body mass index.

The majority of the subjects were girls, aged 10 to 11 years, with non-white skin color, belonging to socioeconomic class C/D/E, whose mothers had at least completed elementary education and a little more than one-third were overweight. The time of physical activity, sedentary behavior and number of breaks the adolescents had was 29.1; 451.0 and 100.3, respectively (Table 1).

In the simple model, there was a significant association between the average number of breaks per day and LDL-c levels (p = 0.030), systolic blood pressure (p = 0.006) and BMI (p <0.001). In the adjusted analysis, only an association between the average number of breaks per day and the BMI (p <0.001) remained statistically significant. Sedentary behavior and BMI did not moderate the association between the number of breaks per day and cardiometabolic markers (Table 2). The final models achieved good quality of fit: absence of multicollinearity (VIF between 1.03 and 3.39), presence of homoscedasticity (Cook-Weisberg test with *p* values ranging from 0.054 to 0.335) and normal distribution in the regression residuals.

**Table 2:** Crude and adjusted linear regression for the association between the average number of breaks per day and cardiometabolic markers in adolescents from João Pessoa, Paraíba, 2014

Variables		Crude			Adjusted†			Sedentary behavior interaction term‡			BMI interaction term§	
	ß	(95%CI)	p	ß	(95%CI)	p	ß	(95%CI)	p	ß	(95%CI)	p
Glucose (mg/dL)	0.011	-0.052; 0.073	0.737	-0.039	-0.138; 0.061	0.446	-0.021	-0.050; 0.009	0.176	-0.003	-0.037; 0.031	0.865
Cholesterol (mg/dL)	-0.181	-0.375; 0.013	0.067	-0.042	-0.336; 0.251	0.778	0.037	-0.051; 0.126	0.404	-0.046	-0.146; 0.055	0.371
Triglycerides (mg/dL)	-0.001	-0.004; 0.002	0.539	0.000	-0.004; 0.005	0.861	0.001	-0.001; 0.002	0.285	0.001	-0.001; 0.002	0.248
HDL-c (mg/dL)	0.040	-0.018; 0.099	0.174	-0.016	-0.104; 0.073	0.729	-0.013	-0.041; 0.014	0.341	0.005	-0.027; 0.036	0.761
LDL-c (mg/dL)	-0.190	-0.362; -0.018	0.030	0.019	-0.238; 0.277	0.882	0.021	-0.058; 0.101	0.598	-0.057	-0.149; 0.034	0.221
SBP (mmHg)	-0.081	-0.139; -0.023	0.006	-0.011	-0.090; 0.068	0.778	0.017	-0.008; 0.041	0.176	-0.005	-0.033; 0.023	0.710
DPB (mmHg)	-0.029	-0.072; 0.014	0.184	-0.026	-0.088; 0.037	0.421	0.008	-0.011; 0.027	0.387	-0.004	-0.025; 0.018	0.739
BMI (kg/m^2^)	-0.051	-0.075; -0.026	0.000	-0.069	-0.102; -0.035	0.000	-0.004	-0.014; 0.006	0.440	--	--	--

ß: beta coefficient; 95% CI: 95% confidence interval; HDL-c: high density lipoprotein; LDL-c: low density lipoprotein; SBP: systolic blood pressure; DBP: diastolic blood pressure; BMI: body mass index;

† Model adjusted for gender, age, skin color, socioeconomic class, mother's level of education, hours of sleep (hours/day), fiber intake (g), lipids (g), saturated fat (g), cholesterol (mg), sodium (mg), time of accelerometer usage (min/day), total moderate to vigorous physical activity (min/day), total sedentary behavior (min/day) and BMI (kg/m^2^), except when it is treated as a dependent variable;

‡ Adjusted model + interaction term between sedentary behavior (<8 hours/day vs. ≥ 8 hours/day) and number of breaks/day;

§ Adjusted model + interaction term between BMI (kg/m^2^) and number of breaks/day.

The results of the Wald test indicated that there were no significant differences in the mean values of cardiometabolic markers between adolescents exposed to ≥ 8 hours of sedentary behavior and <100 breaks / day, ≥ 8 hours of sedentary behavior and ≥ 100 breaks / day, <8 hours of sedentary behavior and <100 breaks / day and <8 hours of sedentary behavior and ≥ 100 breaks / day (Figures 2 and 3).

**Figure 2:** Comparison of the mean values of BMI, systolic and diastolic blood pressure and glucose between combined exposure to sedentary behavior (SB) and breaks (BR) in adolescents, João Pessoa, Paraíba, 2014. ↑ SB = ≥ 8 hours/day; ↓ SB = <8 hours/day; ↑ BR = ≥ 100 breaks/day e; ↓ BR = <100 breaks/day.

**Figure 3:** Comparison of the average values of cholesterol, triglycerides, HDL and LDL between the combined exposure to sedentary behavior (SB) and breaks (BR) in adolescents, João Pessoa, Paraíba, 2014. ↑ CS = ≥ 8 hours/day; ↓ CS = <8 hours/day; ↑ BR = ≥ 100 breaks/day e; ↓ BR = <100 breaks/day.

## Discussion

The results of the present study indicated that the adolescents with the highest number of breaks during sedentary time obtained the lowest BMI values. However, associations with the remaining cardiometabolic markers were not significant and not moderated by the adolescents' nutritional status.

Studies with adults have demonstrated that a larger number of breaks is associated with fewer harmful effects on cardiometabolic health caused by sedentary behavior.^41^ However, in adolescents, it has been associated only with body fat indicators.^2^^,^^6^ The absence of an association between breaks and cardiometabolic markers may be related to the fact that a significant part of the time adolescents spend on sedentary behavior is accumulated in blocks of up to five minutes.^1^^,^^14^^,^^16^ Short sedentary time blocks may minimize the reduced LPL (lipase lipoprotein) enzyme activity and contribute to increased energy expenditure. These two factors are related to the decline in blood glucose and triglycerides and increase in HDL-c levels.^42^

The excessive time in sedentary behavior did not moderate the association between the number of breaks and cardiometabolic markers. An additional analysis showed that more than 80% of the adolescents' sedentary time in the present study was accumulated in intervals of less than 10 minutes, even in those who showed excessive time in sedentary behavior (data not shown in the table). Therefore, it is possible that the benefits of including breaks on cardiometabolic markers are observed in adolescents exposed to long and uninterrupted periods of sedentary behavior.

Some experimental studies have shown that including breaks (moderate to vigorous 3-minute breaks every half hour during three hours of sedentary behavior) reduced insulin, C-peptide^27^^,^^43^ and glucose levels.^43^ However, this result was not confirmed by Saunders et al.^1^ (mild intensity 2-minute breaks every 20 minutes during eight hours of sedentary behavior). The inconsistent results of these studies do not support the hypothesis that the benefits of including breaks occurred in adolescents who spent prolonged periods of time in sedentary behavior.

The possible lower LPL response to the hypotensive effect of sedentary behavior in adolescents and their greater capacity in maintaining cardiometabolic markers close to normal values (homeostasis), when compared to adults, may be other factors that can explain the absence of an association between breaks and cardiometabolic markers in the latter group.

In the present study, adolescents who took more breaks had lower BMI values, reinforcing the findings of other studies.^2^^,^^6^ In terms of clinical relevance, the effect of breaks on BMI showed a low magnitude (for each performed break, a decrease of 0.069 kg/m^2^ in BMI is estimated - effect size = 0.076). Despite this fact, the inclusion of breaks can be an easily implemented practice in the adolescents' life context, and may be one of several actions to be used in interventions aimed at reducing and/or controlling the BMI.

Moreover, breaks during sitting time tends to promote greater energy expenditure, due to the increase in physical activity. In a study with adults, Júdice et al.^15^ observed that a break resulted in an average increase of 1.49 kcal/min in energy expenditure when compared to remaining in the standing position. In adolescents, since breaks may result in energy expenditure similar to that of adults, taking 100 breaks a day would be the equivalent to having a 30-minute walk at moderate intensity.^44^ It has been found that more prolonged sedentary behavior is related to fewer leisure physical activity breaks^45^ and greater consumption of sweets, soft drinks and industrialized/ultraprocessed foods.^46^ As such, adolescents who had more daily breaks could engage in more prolonged leisure physical activity and had a lower intake of these food items. Finally, since this is a cross-sectional study, we cannot exclude the possibility that adolescents with a higher BMI would exhibit more spontaneous movement throughout the day, resulting in fewer breaks in sedentary behavior.

The following are strong points of this study: 1) data were collected from a representative sample of 6^th^ grade-schoolchildren from public schools in a city located in Northeastern Brazil and exhibited sufficient power to test the study hypotheses; different cardiometabolic markers were analyzed and 2) important confounding factors were considered regarding the relationship between sedentary behavior and cardiometabolic markers (physical activity, hours of sleep and food intake).

The following were study limitations: not measuring the adolescents' degree of sexual maturation, a factor that can influence cardiometabolic markers^47^^,^^48^ and some types of sedentary behavior;^49^ reinstating the epoch accelerometer data from 15 to 60 seconds, which could have underestimated sedentary behavior time^50^ and the magnitudes of the associations and the measurement of breaks during sedentary behavior using an accelerometer that measures body acceleration and not postural variation (sitting, reclining, standing).^51^

## Conclusion

Adolescents who had more breaks per day during time in sedentary behavior had lower mean values of BMI but there were no differences regarding the values of the other biochemical cardiometabolic markers (levels of glucose, triglycerides, HDL-c, LDL-c, total cholesterol and blood pressure values), regardless of their nutritional status and excessive exposure to sedentary behavior.
